# Supplementary figures and images for: Identification of APTX disease-causing mutation in two unrelated Jordanian families with cerebellar ataxia and sensitivity to DNA damaging agents
Source: PLoS One. 2020 Aug 4;15(8):e0236808. doi: 10.1371/journal.pone.0236808 (PMC7402469; doi:10.1371/journal.pone.0236808)

Supplementary Fig.1

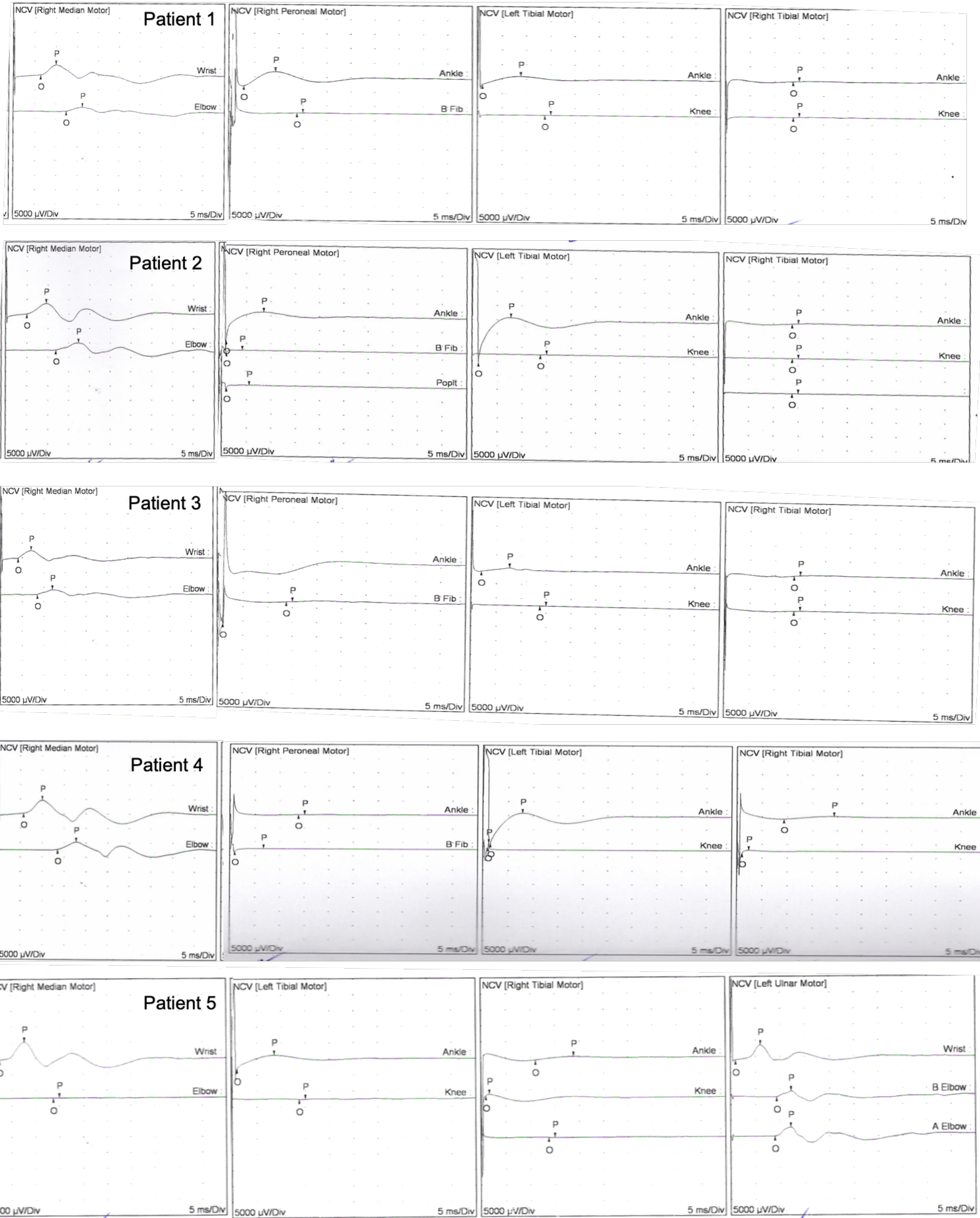

Supplement: S1 Fig — (PDF) [file pone.0236808.s001.pdf]
