# Supplementary material for: Identification of APTX disease-causing mutation in two unrelated Jordanian families with cerebellar ataxia and sensitivity to DNA damaging agents
Source: PLoS One. 2020 Aug 4;15(8):e0236808. doi: 10.1371/journal.pone.0236808 (PMC7402469; doi:10.1371/journal.pone.0236808)
Supplement: S1 Raw images — (PPTX) [file pone.0236808.s002.pptx]

## Slide 1
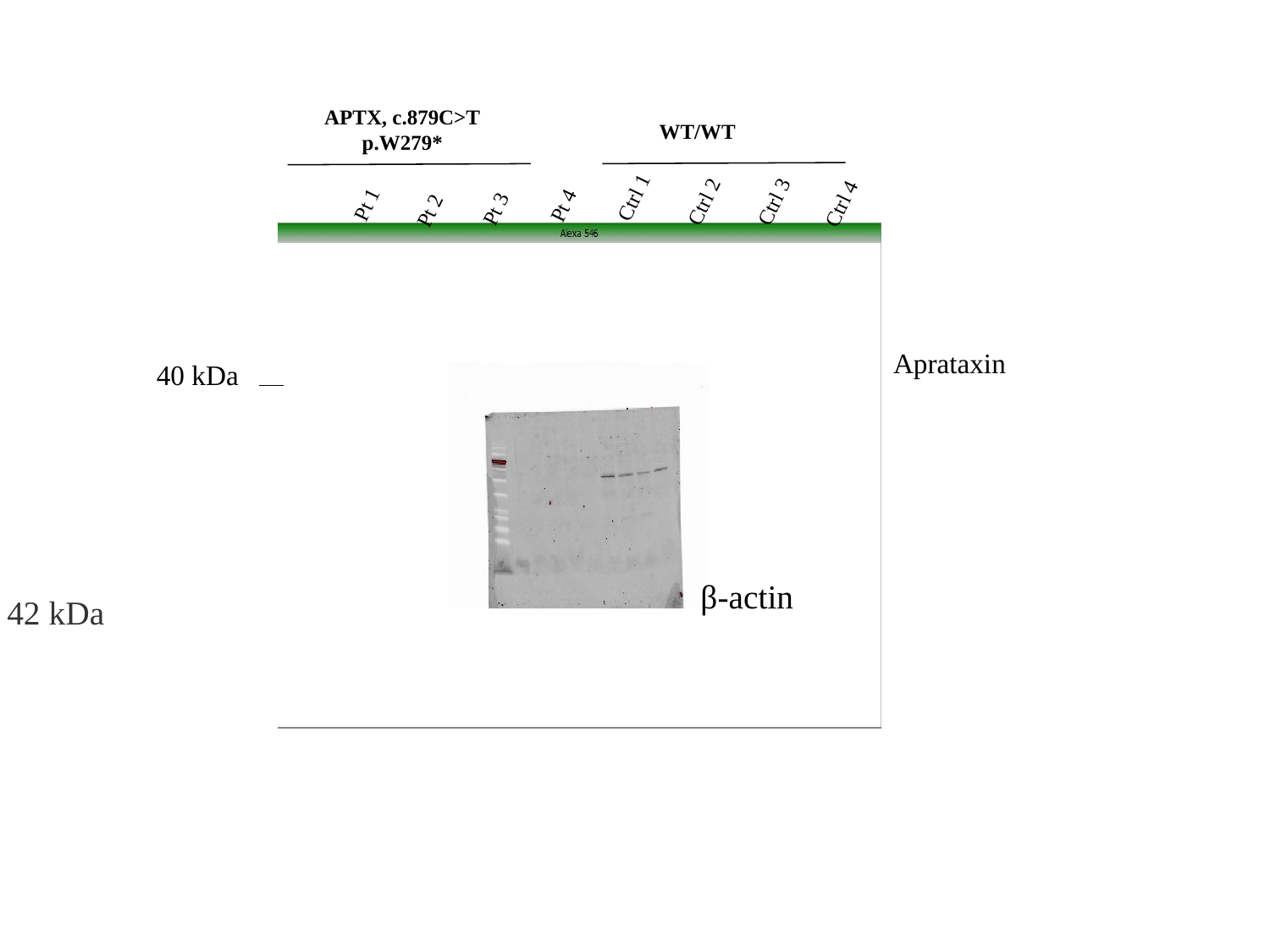

APTX, c.879C>T
p.W279*
WT/WT
Pt 1
Ctrl 1
Pt 4
Pt 3
Ctrl 3
Ctrl 2
Ctrl 4
Pt 2
Aprataxin
40 kDa
β-actin
42 kDa

## Slide 2
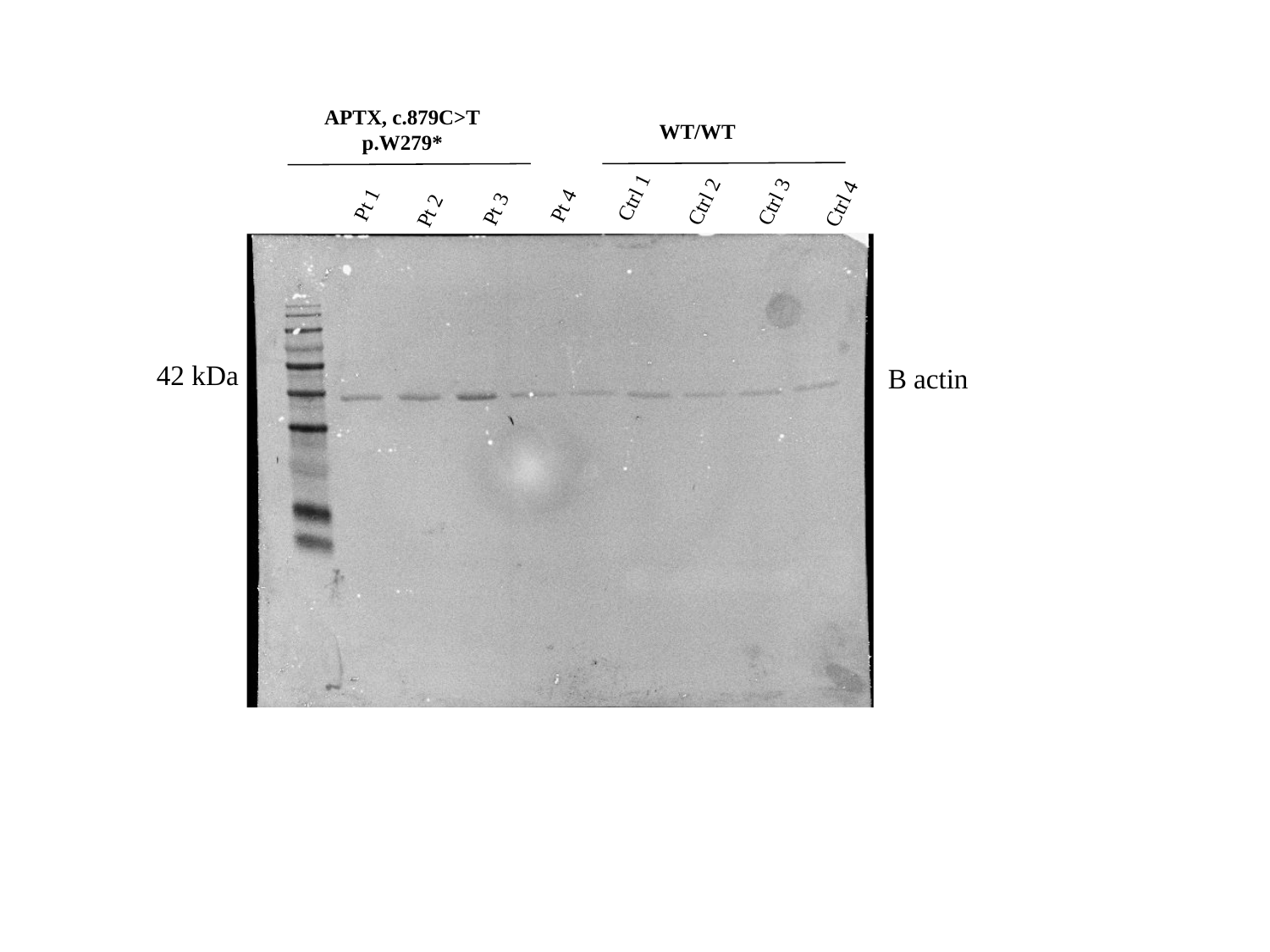

APTX, c.879C>T
p.W279*
WT/WT
Pt 1
Ctrl 1
Pt 4
Pt 3
Ctrl 3
Ctrl 2
Ctrl 4
Pt 2
42 kDa
B actin
