# Supplementary material for: Identification of APTX disease-causing mutation in two unrelated Jordanian families with cerebellar ataxia and sensitivity to DNA damaging agents
Source: PLoS One. 2020 Aug 4;15(8):e0236808. doi: 10.1371/journal.pone.0236808 (PMC7402469; doi:10.1371/journal.pone.0236808)
Supplement: Attachment — Submitted filename: Response to reviewr.docx [file pone.0236808.s003.docx]

**PLOS ONE** PONE-D-20-17895

**Response to Reviewer’s Comments**

Dear Editor,

Regarding our manuscript with Ref. No.: # PONE-D-20-17895 entitled “Identification of APTX disease-causing mutation in two unrelated Jordanian families with cerebellar ataxia and sensitivity to DNA damaging agents.’’ We are extremely grateful to the reviewers for their positive comments on this manuscript. All changes to the manuscript have been tracked on the revised submission. We have addressed each comment individually below. Please also find attached marked and unmarked versions of our manuscript. The changes are directly tracked in the “marked” document.

Sincerely, and on behalf of the co-authors

Corresponding author,

Nidaa A Ababneh

**Response to Editor:**

Comment 1.  Whole exome sequencing for identification of mutations in one gene seems excessive.  The rationale for this approach should be better described and included in the Discussion.

**Response:** Thank you. The rationale has been described in the discussion and highlighted in yellow. Line: 333

In this study, WES was used to assist in the diagnosis of an unidentified cerebellar ataxia in two unrelated families with high rate of consanguinity. Currently, WES has emerged as a more straightforward and cost-effective approach to screen pathogenic variants rather than using other traditional molecular diagnostic techniques.

Based on our filtering criteria and the recommendation of the American College of Medical Genetics and Genomics and the Association for Molecular Pathology (20), we identified a recurrent variant of AOA1 in Jordanian families. This *APTX* truncating mutation p.W279* was found in 22 individuals of 2 unrelated families, family A (n=18) and family B (n=4). Early-onset slowly progressive cerebellar ataxia, was found to be the presenting symptom in the majority of our cases described in this study.

Comment 2. Address reviewer 1's points regarding the inconsistencies in the patient descriptions from line 229 and Figure1.

**Response:** we have modified the patient descriptions as requested. The reviewer’s points have been addresses and tracked in the same position.

Comment 3.   Include more information in the Discussion on the impact of the results from this study.

**Response:** Thank you for your suggestion. We have added some data to the discussion as requested. Please see Line: 404 highlighted in yellow.

Comment 4. Address reviewer 2's comments about treatment of the cell lines.

**Response:** thank you, the comments have been addressed as requested.

Comment 5.  Ensure that mutation nomenclature is accurate.  (See Reviewer 1's comments).

**Response:** we have checked the mutation nomenclature and made the required correction.

Comment 6.  Ensure that all gene names are italicized.

**Response:** All gene names have been changed to italic format.

Comment 7.  Include a link to the exome data as all data needs to be available.

**Response:** we have provided a link at the end of the manuscript; line: 441

Comment 8.  PLOS ONE now requires that submissions reporting blots or gels include original uncropped blot/gel image data as a supplement or in a public repository. This needs to be added to supplementary material.

**Response:** All uncropped blots have been added as supplementary material in PowerPoint document.

Comment 9.  As one of the authors is affiliated with Prevention Genetics this needs to be included in the conflicts of interest section.

**Response:** Please note that the author ‘Belal Azab’ was affiliated to the University of Jordan during the time of the study. He has recently moved to the Prevention Genetics and there is NO COFLICT of interest to declare. He removed the prevention Genetics affiliation and he only kept the academic affiliations.

Comment 10. Address other points raised by the reviewers.

**Response:** Done, please find our point-by-point response in below**.**

Comment 11: We note that you have provided funding information that is not currently declared in your Funding Statement. However, funding information should not appear in the Acknowledgments section or other areas of your manuscript. We will only publish funding information present in the Funding Statement section of the online submission form.

Please remove any funding-related text from the manuscript and let us know how you would like to update your Funding Statement. Currently, your Funding Statement reads as follows:

"The author(s) received no specific funding for this work."

**Response:** Please kindly note that we have removed the funding information from the Acknowledgements sections. Please update our funding statement as the following: This work was supported by the deanship of scientific research, University of Jordan, grant (2018-2017/2). The funding organization did not play a role in the study design, data collection and analysis, decision to publish, or preparation of the manuscript and only provided financial support in the form of research materials.

3. Thank you for stating the following in the Competing Interests section:

"The authors have declared that no competing interests exist."

We note that one or more of the authors are employed by a commercial company: Prevention Genetics.

3.1 Please provide an amended Funding Statement declaring this commercial affiliation, as well as a statement regarding the Role of Funders in your study. If the funding organization did not play a role in the study design, data collection and analysis, decision to publish, or preparation of the manuscript and only provided financial support in the form of authors' salaries and/or research materials, please review your statements relating to the author contributions, and ensure you have specifically and accurately indicated the role(s) that these authors had in your study. You can update author roles in the Author Contributions section of the online submission form.

**Response:** Please note that the author ‘Belal Azab’ was affiliated to the University of Jordan during the time of the study. He has recently moved to the Prevention Genetics and there is NO COFLICT of interest to declare. He removed the prevention Genetics affiliation and he only kept the academic affiliations.

**Response to Reviewer’s comment**
**Reviewer #1:** Ababneh et al described a case of AOA1. This is the first case of the Jordanian.

Minor comments

L119 FBC should be fully spelled out at first appearance.

**Response:** the change has been made as requested. FBS indicates the full blood count.

L229 All patients had typical symptoms of cerebellar ataxia with disease onset at age of 3-7 years, clinical evidence of cerebellar ataxia, severe sensorimotor peripheral neuropathy with distal muscle wasting and weakness affecting predominantly the lower limbs and chorea (Fig 1A, Table 1) Fig 1A is the familial pedigree tree. The sentence described above does not match the Fig 1A.

**Response:** We agree with the reviewer’s comment. Fig 1A has been removed from this section.

L236 albumin levels were elevated in 10 patients (45.5%). In tables 1, the author described as hypoalbuminemia. Usually, in AOA1, serum albumin level is decreased.

**Response:** Absolutely true, the albumin levels were decreased in 10 patients. Apologise for the typing error, corrected and tracked directly on the manuscript.

L236 AFP levels were observed in eight patients 236(36.4%). Does AFP increase or decreased? In tables 1, the author described as increased AFP. Usually in AOA1, serum AFP level is normal.

**Response:** Although other studies reported normal serum AFP level, we observed a slight elevation in AFP levels in 8 patients.

L261 APTX p.GW2 79* → APTX p. W279*

**Response:** We apologise for the typing mistake. Please kindly note that the mutation nomenclature has been corrected and highlighted in yellow.

**Reviewer #2:** The authors described a reported mutation, pW279* in Jordan patients with AOA1. Fibroblasts from 6 patients were used.
The paper is well written, but no novel finding is described, except for the first report from Jordan. In addition, there are several points to be addressed.

Major points:

1. The result of MMC, MMS, and H2O2 treatment is not new, with some discrepancies between the present results and the reported ones. The authors described that the discrepancies were attributed to a different mutation. How such mutational differences affect the result is important.

**Response:** In response to the reviewer’s comment, the position of the mutation has an effect on protein stability. Most mutations occur in either the Histidine Triad domain or zinc finger nuclease domain, this leads to either destabilization or truncation of the protein. The exact location of the mutation and its type as missense or nonsense can affect the protein structure, particularly the catalytic function of the protein. Thus, resulting in a wide range of phenotypes and in some cases could result in less or more sever clinical symptoms (further clarification in the discussion has been added).

2. Interesting thing is therapy, since the described mutation p.W279* is a common mutation in AOA1. Please describe that prevention of cell death from the genotoxins.

**Response:** Treatment with antioxidants lead to decrease ROS‐induced SSBs might reduce cell death induced by genotoxic agents, such cytoprotection with antioxidants has been reported to provide clinically substantial benefits (Cooper et a., 2003). If this has been confirmed, it will support the idea that the pathomechanism in AOA1 is the result of limited repair capacity of ROS‐induced SSBs.

 Cooper JM, Schapira AH. Friedreich's ataxia: disease mechanisms, antioxidant and coenzyme Q10 therapy. Biofactors 2003; 18: 163–171.

3. If proteasome inhibitors, such as lactascystin, are used, the truncated protein p.W279* would be detectable?

**Response:** Thank you for your suggestion. Please note that the western blotting was performed on the physiological protein levels without any treatment. However, we can try your suggestion in the near future.

Minor points:
1. The human gene name should be in italicized, ex APTX.

**Response:** please kindly note that we have changed all *APTX* gene names to italic format, directly tracked on the manuscript.

2. Figure legends were not present.

**Response:** Please kindly note that the figure legends are present in the results part, tracked and highlighted in green.

While revising your submission, please upload your figure files to the Preflight Analysis and Conversion Engine (PACE) digital diagnostic tool, <https://pacev2.apexcovantage.com/>. PACE helps ensure that figures meet PLOS requirements. To use PACE, you must first register as a user. Registration is free. Then, login and navigate to the UPLOAD tab, where you will find detailed instructions on how to use the tool. If you encounter any issues or have any questions when using PACE, please email PLOS at [figures@plos.org](mailto:figures@plos.org). Please note that Supporting Information files do not need this step.
